# Carbon mineralization pathways and bioturbation in coastal Brazilian sediments

**DOI:** 10.1038/srep16122

**Published:** 2015-11-03

**Authors:** Cintia O. Quintana, Maurício Shimabukuro, Camila O. Pereira, Betina G. R. Alves, Paula C. Moraes, Thomas Valdemarsen, Erik Kristensen, Paulo Y. G. Sumida

**Affiliations:** 1 Instituto Oceanográfico da Universidade de São Paulo, Praça do Oceanográfico, 191, Cidade Universitária, 05508-120, São Paulo, Brazil; 2 Department of Biology, University of Southern Denmark, Campusvej 55, Odense M, 5230, Denmark

## Abstract

Carbon mineralization processes and their dependence on environmental conditions (e.g. through macrobenthic bioturbation) have been widely studied in temperate coastal sediments, but almost nothing is known about these processes in subtropical coastal sediments. This study investigated pathways of organic carbon mineralization and associated effects of macrobenthic bioturbation in winter and summer (September 2012 and February 2014) at the SE Brazilian coast. Iron reduction (FeR) was responsible for 73–81% of total microbial carbon mineralization in September 2012 and 32–61% in February 2014. Similar high rates of FeR have only been documented a few times in coastal sediments and can be sustained by the presence of large bioturbators. Denitrification accounted for 5–27% of total microbial carbon mineralization while no SO_4_^2−^ reduction was detected in any season. Redox profiles suggested that conditions were less reduced in February 2014 than in September 2012, probably associated with low reactivity of the organic matter, higher rates of aerobic respiration and bioirrigation by the higher density of small-macrofauna. Bioturbation by small macrofauna may maintain the sediment oxidized in summer, while large-sized species stimulate the reoxidation of reduced compounds throughout the year. Therefore, bioturbation seems to have an important role modulating the pathways of carbon mineralization in the area.

Carbon (C) cycling in coastal ecosystems is controlled by microbial processes in the sediment, where most of the organic carbon is mineralized aerobically and anaerobically into CO_2_ and nutrients (i.e. ammonium, nitrate and phosphate). In a balanced coastal ecosystem, organic C is mineralized in the sediment under optimal biogeochemical conditions, while maintaining redox levels that allows a diverse benthic flora and fauna. The nutrients returned from the sediment to the water column may participate in new primary production^1^, which may be beneficial in nutrient limited areas. The rate of organic C mineralization in the sediment is controlled primarily by the organic matter input and properties (i.e. quantity, composition, reactivity), environmental conditions (i.e., sediment type, temperature, salinity, currents), availability of electron acceptors and bioturbation activity^2^,^3^.

In temperate coastal sediments, aerobic respiration and anaerobic sulfate reduction are assumed to roughly contribute with 50% each to total C mineralization^4^. In organic-rich areas including intertidal flats and eutrophic coastal lagoons, O_2_ penetrates only few mm into the sediment and sulfate reduction may account for up to 80% of total C mineralization^4^. Other anaerobic processes, such as denitrification and iron respiration, may be important for C oxidation in certain continental shelf areas^5^. Manganese respiration seems to be constrained to Mn-oxide rich sediments, where it may account for 90% of anaerobic C mineralization^5^.

Limited information is available on biogeochemical processes in coastal Brazilian sediments. The largest pool of the sediment organic C is labile in these areas, since the input is primarily marine and derived from diatoms, phytoflagellate and zooplankton blooms associated to upwelling and sediment resuspension events^6^,^7^,^8^. The microbial community has a temporal biomass variation following phytoplankton blooms and experiments with homogenized sediment have confirmed that microbial density increases markedly 24–48 h after the deposition of diatoms and phytoflagellates at the sediment surface^8^,^9^. This suggests that the sediment microbial community is dynamic and that microorganisms rapidly consume and degrade any deposited labile organic C. However, very little is known about microbial reaction rates and pathways of C mineralization.

Coastal sediments usually have a high abundance of burrowing benthic macrofauna^4^. The bioturbation activities of these organisms transport electron acceptors and labile organic C between oxic and anoxic zones of the sediment by burrow ventilation and particle reworking, which often accelerates the processes of organic C oxidation considerably^2^. The oxidized burrow walls resulting from bioirrigation (i.e. diffusion of oxidized solutes from burrow water) create microenvironments with steep gradients between reduced and oxidized compounds. These transition zones support increased microbial activities providing ideal conditions for reoxidation processes^10^. Important biogeochemical C oxidation reactions such as denitrification, Mn and Fe reduction are highly dependent on reoxidation and transport processes associated to bioturbation^11^,^12^. Although the dynamics of macrobenthic communities is relatively well known in coastal Brazilian sediments^13^,^14^, their bioturbation potential and effects on benthic C oxidation processes have never been quantified.

The aim of this study was to investigate total C mineralization, partitioning of dominating heterotrophic processes and the role of macrobenthic bioturbation in coastal Brazilian sediments during two contrasting seasons, i.e. winter and summer (September 2012 and February 2014, hereafter referred as September and February). Sediment cores collected at three stations from 5–12 m water depth were incubated 3–5 days in the laboratory for measurements of solute fluxes across the sediment-water interface, bioirrigation, sediment redox conditions, porewater chemistry and solid-phase sediment characteristics. Additional sediment cores were sampled for anoxic sediment incubations, and quantification and identification of benthic macrofauna. A budget combining flux measurements, anaerobic incubations and metabolism of the macrobenthic community was used to assess microbial pathways involved in organic C oxidation.

## Results

### Bottom water O_2_, sediment characteristics and pigments

Bottom water O_2_ was partly depleted in September with lower concentrations (117–120 μM) than in February (200–213 μM) (F_1,16_ = 393.8, p < 0.001) (Supplementary Table S1). Sediment characteristics (i.e. density and porosity) were similar among stations and months, although with somewhat lower porosity in February (0.59–0.66) than September (0.70–0.74). Annual average of total organic carbon (TOC) and total nitrogen (TN) measured previously on the same stations ranged between 1–2% and 0.2%, respectively^8^. Median grain size varied from 13 to 58 μm with 23–83% silt+clay and tended to increase with water depth^8^. Sediment chlorophyll-a content in the upper 3 cm was higher in September (1.0–2.5 μg g^−1^) than in February (1.0–1.2 μg g^−1^) (F_1, 16_ = 19.4, p = 0.001) with St 5 reaching the highest levels (2.5 ± 0.2 μg g^−1^) (Supplementary Table S1). The corresponding phaeopigment concentrations were also significantly higher in September (3.4–4.5 μg g^−1^) than in February (2.4–2.9 μg g^−1^) (F_1,16_ = 21.3, p < 0.001), but similar among stations (Supplementary Table S1).

### Fluxes of TCO_2_, O_2_ and nutrients

The average TCO_2_ efflux was significantly lower in September (19–23 mmol m^−2^ d^−1^) than in February (31–34 mmol m^−2^ d^−1^) (F_1,56_ = 31.6, p < 0.001; Table 1, Supplementary Table S1). A similar but more pronounced pattern was observed for O_2_ consumption with rates in February ranging from −32 to −35 mmol m^−2^ d^−1^ and in September from −9 to −12 mmol m^−2^ d^−1^ (F_1,56_ = 319.0, p < 0.001; Table 1). There was no significant difference in TCO_2_ efflux and O_2_ consumption among stations (Table 1, Supplementary Table S1). NH_4_^+^ exchange ranged from consumption in September (−0.1 to −1.2 mmol m^−2^ d^−1^) to release in February (1.1 to 1.5 mmol m^−2^ d^−1^), but rates were not significantly different among stations and months (Table 1). Conversely, NO_x_^−^ fluxes were significantly different between September (−0.4 to −0.6 mmol m^−2^ d^−1^) and February (−0.1 to 0.2 mmol m^−2^ d^−1^) (F_1,36_ = 118.2, p < 0.001) (Table 1, Supplementary Table S1). PO_4_^3−^ flux was not detected at any stations neither in September nor February.

### Vertical redox profiles

**Figure 1:**
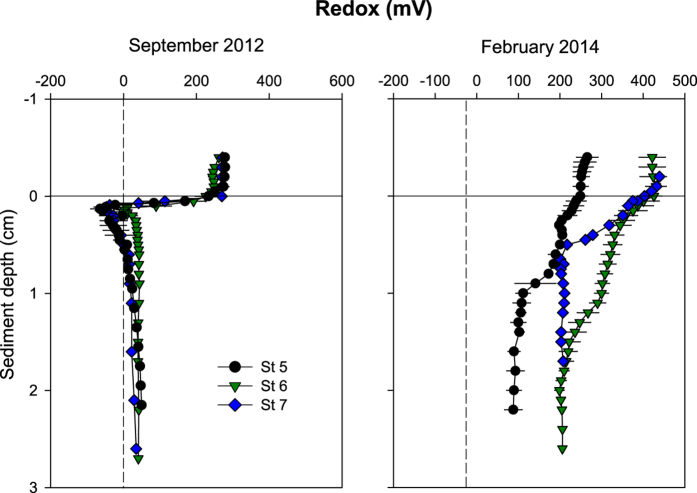
Redox profiles in September 2012 and February 2014 at St 5, St 6 and St 7. Values represent mean ± standard error (n = 3–4).

Redox profiles were consistently different between September and February reflecting temporal changes in sediment conditions (Fig. 1). The profiles were steepest in September and similar among stations, with a distinct discontinuity just below the sediment surface (Fig. 1). Depth of the oxidized zone, i.e. Eh > 0 mV, was narrow and ranged from 0.07 mm at St 5 and St 7 to 0.15 mm at St 6. Redox was most negative with values of −64 and −47 mV at St 5 and St 7, respectively and −1 mV at St 6 at 1.1–1.6 mm depth. Below this negative peak, Eh increased to positive values, reaching 9–50 mV. Redox conditions were more oxidized in February and negative Eh was not detected (Fig. 1). Redox instead decreased gradually with sediment depth. Thus, St 5 had Eh < 200 mV below 3 mm depth while the same Eh level was reached at 2.0 and 0.6 mm depth at St 6 and St 7, respectively (Fig. 1).

### Microbial reaction rates

**Figure 2:**
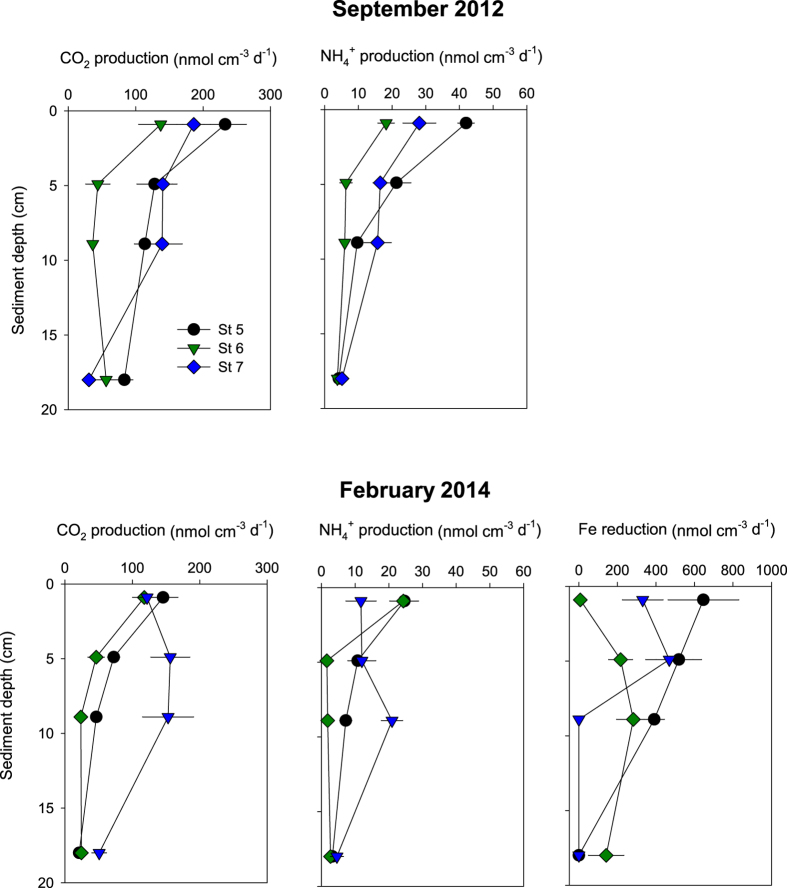
Microbial reaction rates in September 2012 and February 2014 at St 5, St 6 and St 7. Values represent mean ± standard error (n = 3).

The anoxic jar incubations showed that microbial TCO_2_ production in September decreased from 138–233 nmol cm^−3^ d^−1^ at the sediment surface to 31–83 nmol cm^−3^ d^−1^ at 16–18 cm depth (Fig. 2). Rates of TCO_2_ production at St 5 and St 6 followed the same decreasing depth pattern in February with rates from 118–145 nmol cm^−3^ d^−1^ at 0–2 cm to 21–24 nmol cm^−3^ d^−1^ at 16–18 cm depth (Fig. 2). TCO_2_ production at St 7, on the other hand, increased with depth reaching a maximum of 153–156 nmol cm^−3^ d^−1^ at 4–10 cm. NH_4_^+^ adsorption coefficients varied from 0.61 to 0.87. NH_4_^+^ production corrected for adsorption decreased in a similar pattern with depth irrespective of season and station from 18–42 nmol cm^−3^ d^−1^ at the surface to 3–5 nmol cm^−3^ d^−1^ at 16–18 cm depth, except for St 7 in February, where the highest NH_4_^+^ production of 21 nmol cm^−3^ d^−1^ was evident at 8–10 cm depth (Fig. 2). The depth integrated TCO_2_ and NH_4_^+^ production were not significantly different among stations and time. Fe reduction (FeR) in February reached high rates, particularly in the upper 10 cm at St 5 (391–646 nmol cm^−3^ d^−1^) (Fig. 2). St 5 and St 7 exhibited highest FeR rates of 216–469 nmol cm^−3^ d^−1^ at 0–10 cm depth. SO_4_^2−^ reduction was not detected at any of the stations during September or February.

### Porewater solutes

**Figure 3:**
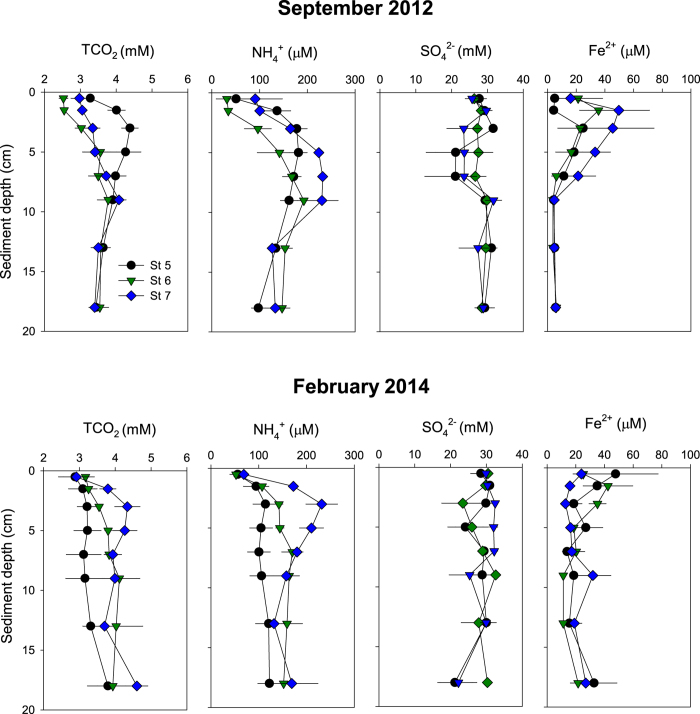
Vertical porewater profiles of solutes in September 2012 and February 2014 at St 5, St 6 and St 7. Values represent mean ± standard error (n = 3).

Porewater TCO_2_ increased steeply in the upper 3 cm at St 5 in September and at St 7 in February, reaching a maximum of 4.3–4.4 mM (Fig. 3). At the other stations, TCO_2_ increased gradually from ~3 mM in the surface to ~4 mM at 18 cm depth. Porewater NH_4_^+^ showed the same overall pattern as TCO_2_ and increased from 32–91 μΜ at the surface to 114–232 μΜ at 5–10 cm depth with highest concentrations at St 7 in both September and February (Fig. 3). Furthermore, the near-surface NH_4_^+^ profiles at St 7 were steeper in February than in September. NH_4_^+^ decreased gradually below 4–8 cm sediment depth at all stations (Fig. 3). Porewater NO_x_^−^ and PO_4_^3−^ concentrations were generally very low varying from 1 to 4 μM and 4 to 21 μM, in September and February respectively (data not shown). Similarly, porewater SO_4_^2−^ varied little among the sediment intervals, stations and months (21–30 mM) (Fig. 3). Subsurface peaks of dissolved Fe^2+^ were evident in September with concentrations up to 35–50 μΜ at 2–3 cm depth decreasing to almost zero below 8 cm depth (Fig. 3). In February, Fe^2+^ levels varied between 11 and 48 μΜ without any clear depth pattern (Fig. 3). Although the porewater profiles in general varied in space and time, the depth integrated TCO_2_, nutrients, SO_4_^2−^, and Fe^2+^ inventories were not significantly different.

### Solid-phase of reactive Fe(II) and Fe(III)

**Figure 4:**
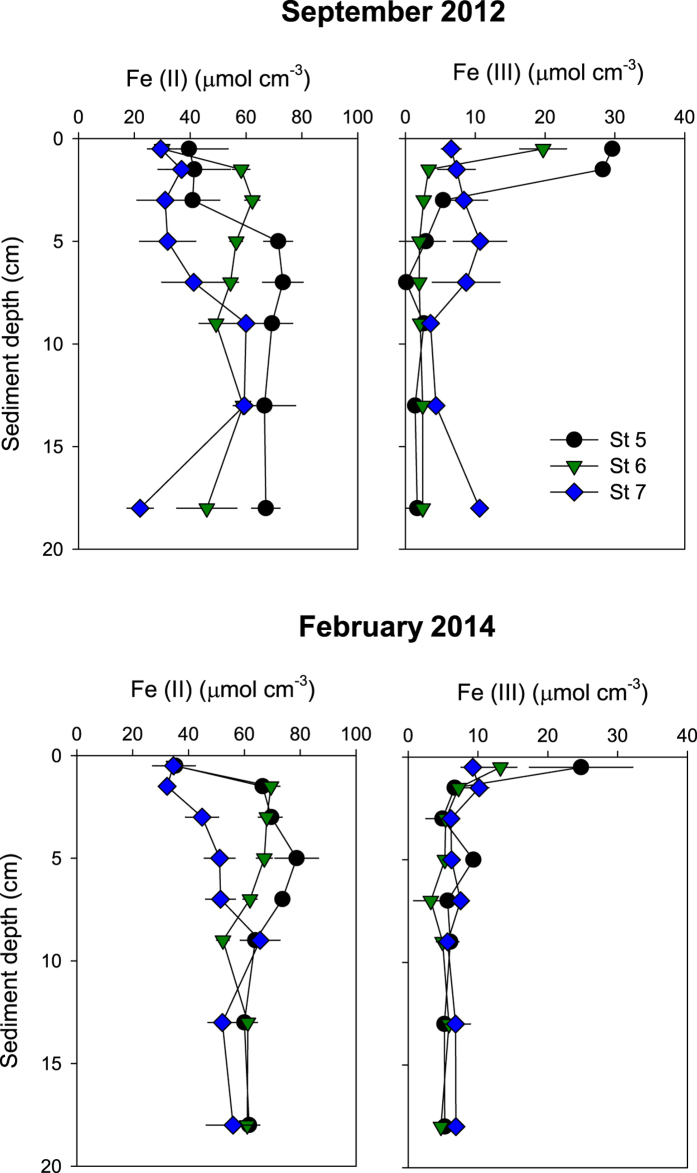
Solid phase reactive Fe(II) and Fe(III) in September 2012 and February 2014 at St 5, St 6 and St 7. Values represent mean ± standard error (n = 3).

Vertical profiles of solid phase Fe(II) and Fe(III) showed similar patterns at the three stations during September and February (Fig. 4). Fe(II) varied from 30–35 μmol cm^−3^ at the surface to 69–78 μmol cm^−3^ at 4–8 cm depth, but with the consistently lowest levels at St 7 from 2 to 8 cm depth (30–51 μmol cm^−3^). Fe(II) was 1–2 fold higher than Fe (III) near the sediment surface (Fig. 4), except for a 4 fold difference at St 7, where Fe(III) was low at 0–1 cm depth (7–9 μmol cm^−3^). Fe(III) decreased sharply from 13–30 μmol cm^−3^ near the surface to <5 μmol cm^−3^ below 2 cm depth at St 5 and 6. The depth integrated Fe(II) and Fe(III) concentrations were not significantly different among stations and months.

### Macrofaunal community structure, metabolism and bioirrigation

Macrofaunal abundance was significantly higher in February (1297–1777 ind m^−2^) than in September (96–192 ind m^−2^) (F_1,17_ = 48.5, p < 0.001, Table 2). There was no significant difference in macrofaunal abundance between stations in either month (Supplementary Table S1). The community consisted of only 7 species in September, including the polychaetes *Ninoe brasiliensis* and *Glycera lapidum*, the bivalve *Nucula semiornata*, small unidentified crustaceans, the ophiuroid Amphiuridae sp. and the hemichordate Enteropneusta sp. The benthic fauna was more diverse in February and consisted of 27 species with dominance of small polychaetes *Prionospio dayi*, *Magelona posterolongata*, paranoids and capitellids, small crabs of the genus *Pinnixa*, the amphipods *Photis longicaudata* and *Ampelisca paria*, the bivalve *Ctena pectinata*, and the hemichordate Enteropneusta sp. The density of epifaunal (F_1,17_ = 17.8, p = 0.001), surface (F_1,17_ = 11.8, p = 0.005) and gallery diffusors (F_1,17_ = 17.8, p = 0.001) was significantly higher in February (96 to 1008 ind m^−2^) than in September (0 to 144 ind m^−2^) (Supplementary Table S1). Upward and downward conveyors only occurred in February with densities varying from 48–288 ind m^−2^.

The estimated biomass (ash free dry weight: AFDW) of macrofauna was significantly higher in February (245–3029 mg m^−2^) than in September (32–402 mg m^−2^) (F_1,17_ = 6.3, p = 0.03, Table 2, Supplementary Table S1). The average biomass per individual was 1.5 ± 0.7 mg AFDW in September and 0.9 ± 0.7 mg AFDW in February. Accordingly, the estimated macrofaunal community respiration was 1–7 mmol m^−2^ d^−1^ in September and 8–10 mmol m^−2^ d^−1^ in February (Table 2).

**Figure 5:**
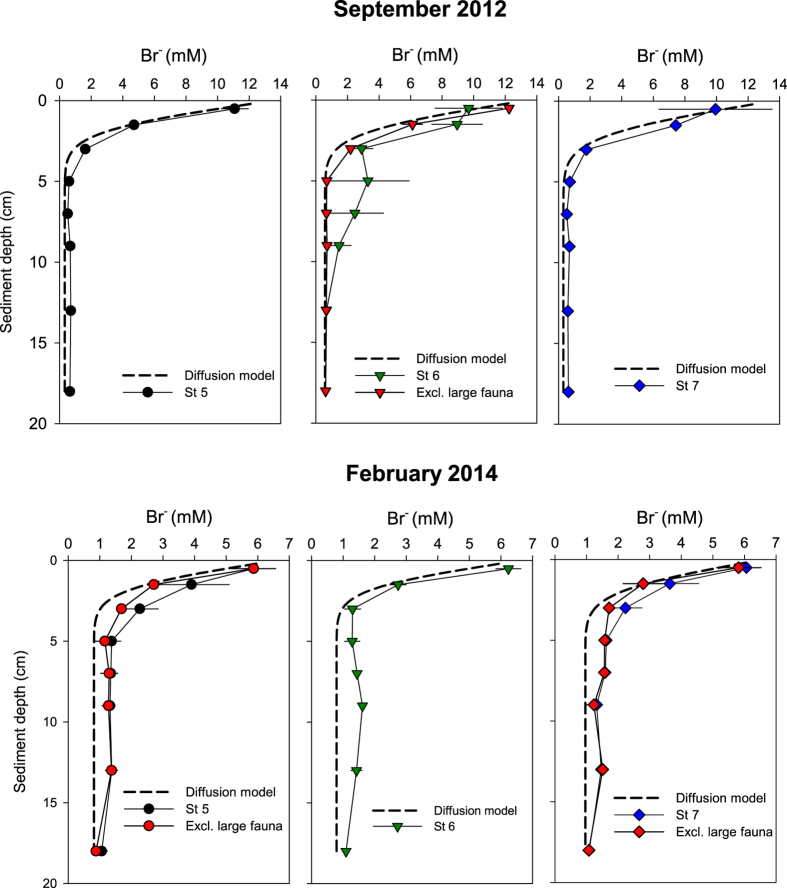
Bromide profiles in September 2012 and February 2014 at St 5, St 6 and St 7. Red symbols represent bromide profiles excluding the effect of large fauna. Values represent mean ± standard error (n = 3).

The Br^−^ concentration in the overlying water was on average 13.5 mM in September and 7.0 mM in February. The modeled diffusive Br^−^ profiles without fauna decreased rapidly with depth reaching background values of 0.3–1.0 mM at around 3–4 cm (Fig. 5). The community bioirrigation estimated from the excess Br^−^ inventory in the sediment varied from 4 to 9 L m^−2^ d^−1^ in September and from 8 to14 L m^−2^ d^−1^ in February (Fig. 5, Table 2) with no significant difference between seasons. The presence of the large Enteropneusta sp. (~1 g individual wet weight) in few cores (i.e. one at St 6 in September and one at St 5 and St 7 in February) probably masked the effect of the smaller-sized fauna, i.e. <1 g wet weight (Fig. 5, Table 2). An extra analysis excluding cores with large fauna revealed significantly higher small-fauna bioirrigation rates in February than September (F_1,15_ = 13.6, p = 0.004, Table 2, Supplementary Table S1).

## Discussion

Rates of TCO_2_ efflux across the sediment-water interface provide a measure of total benthic aerobic and anaerobic C oxidation, while the depth integrated TCO_2_ production measured in jars determines anaerobic C oxidation in the examined depth interval. TCO_2_ efflux and anaerobic TCO_2_ production measured in sediment from the coast of Ubatuba are ca. 2-fold lower than the rates recorded in intertidal areas, but comparable with rates from deeper continental shelf areas, reflecting the meso-oligotrophic nature of the study area^5^,^15^. In contrast to other coastal areas where sulfate reduction accounts for 50–80% of total C oxidation^4^, there was a complete lack of sulfate reduction in anaerobic sediment incubations from Ubatuba, suggesting that this process was hampered by the presence of thermodynamically more favorable electron acceptors, e.g. O_2_, NO_3_^−^, and metal-oxides^5^,^15^.

The partitioning of microbial respiration pathways is determined from TCO_2_ budgets based on fluxes, benthic fauna metabolism and depth integrated jar rates, assuming that any contribution to fluxes from C-oxidation below 20 cm can be ignored. TCO_2_ effluxes are an integrated measure of total C mineralization, including fauna metabolism, and aerobic and anaerobic microbial processes (Table 3). Assuming RQ (TCO_2_ production/O_2_ consumption) = 1 for benthic fauna metabolism, we estimate that the TCO_2_ production by the total macrobenthic community contributed 5–32% to TCO_2_ effluxes (Tables 2 and 3). The remainder is therefore microbial C mineralization (Table 3). Anaerobic C mineralization (i.e. TCO_2_ production in jars) is used to obtain the relative contribution of various anaerobic processes. Anaerobic processes that contribute to the TCO_2_ production in cores and jars were identified as denitrification, iron reduction, and other anaerobic processes (e.g. manganese reduction) (Table 3).

Depth integrated C-oxidation by iron reduction (FeR) as measured in jars accounted for 9–16 mmol C m^−2^ d^−1^ (i.e. converted to C equivalents using the ratio 1/4) in February. The unfortunate lack of FeR measurements in September is compensated by applying the relationship between the concentration of reactive Fe(III) in the sediment and relative contribution of FeR to total anaerobic C mineralization^16^. Thus, the estimated depth integrated C-oxidation by FeR in September varies from 12 to 25 mmol C m^−2^ d^−1^, which is somewhat, but not significantly, higher than the measured FeR in February. Such difference was expected because Fe(III) levels were higher in September than in February. The estimated FeR based on the same relationship in February ranged between 8–21 mmol C m^−2^ d^−1^ and is comparable to the FeR levels measured in jar experiments, confirming that this approach is valid for Ubatuba sediments. FeR generally accounted for 90–100% of anaerobic C mineralization (Table 3). FeR was also the process driving most (73–81%) of total microbial C mineralization in September, whereas lower contribution was evident in February (32–61%, Table 3), where fauna and aerobic processes accounted for a greater proportion of total benthic metabolism. The volume specific FeR rates obtained here are comparable to the highest recorded in marine sediments, such as Kattegat, Amazon inner shelf and Indonesian coast^16^,^17^,^18^. Contribution of FeR >70% to anaerobic processes has been registered in Kattegat, Artic shelf and intertidal mangrove sediments^15^,^16^,^19^, but is here documented for the first time in an area representative for most of the Southeastern Brazilian coastal areas, i.e. small enclosed bays surrounded by granitic mountains^20^,^21^.

The consistently high FeR irrespective of season indicates that the pool of reactive Fe(III) was constantly replenished throughout the year. Reoxidation of Fe(II) typically occurs by transport of Fe(II) to interfaces where O_2_, NO_3_^−^ or Mn(IV) are present. Both the transport of iron and reoxidation is enhanced in the presence of benthic fauna. The deep burrowing Enteropneusta sp. was present in Ubatuba during both September and February, which implies that this animal may have an important role for replenishing Fe(III) down to 14–20 cm sediment depth. The average abundance of Enteropneusta sp. and other deep and large burrowing species including bivalves, sipunculids and ophiuroids varied from 0 to 48 ind m^−2^ and is within the range observed before (7 to 36 ind m^−2^)^14^,^22^. Similar densities of other large fauna such as the polychaete *Arenicola marina* and fiddler crabs *Uca* sp. in estuarine and mangrove areas, respectively, can significantly increase Fe(III) content and stimulate Fe reduction in the sediment^11^,^12^. The role of benthic fauna and in particular large burrowers for downward transport of solutes (e.g. oxygen) is clearly evident in this study from the Br^−^ profiles and the high bioirrigation rates with large fauna (8–12 L m^−2^ d^−1^) (Table 2, Fig. 5), which are comparable to or higher than previously recorded in coastal areas^23^,^24^. The associated bioturbation effects of large fauna may have even higher impact on solute transport, reoxidation and organic carbon mineralization than measured here, since their abundances were probably underestimated by the relatively small sampling unit used in this study (0.008 m^2^)^25^. The contribution of Fe oxides derived from sedimentation for FeR is less important in areas with intense bioturbation of large fauna, but external sources contribute to the long-term enrichment of the sediment with sufficient amounts of reactive iron. In Ubatuba Bay, the deposition of resuspended particles after the fall-winter stormy season and increased river runoff after summer rain may be a major contributor of reactive iron^26^.

Denitrification (DENIT) can be estimated based on the C:N stoichiometry of TCO_2_ and NH_4_^+^ production measured in jars^27^. Total N production is calculated by dividing the microbial C mineralization (i.e. TCO_2_ effluxes minus fauna metabolism) with the C:N ratio found in jars (Supplementary Table S2). The reliability of the jar-based C:N ratios of mineralized organic C and N (6–9) is confirmed from the C:N slope (5–9) of linear regressions of TCO_2_ and NH_4_^+^ porewater profiles in the upper 10 cm sediment when considering cores as open and diffusion dominated systems^28^. DENIT can then be estimated as the missing N between the total N mineralization and DIN (= NH_4_^+^ + NO_x_^−^) fluxes. Accordingly, DENIT varies from 1 to 4 mmol m^−2^ d^−1^ in September and February (Supplementary Table S1). DENIT (i.e. converted to C units by 5/4 ratio) was therefore responsible for 5–27% of total C mineralization in both periods (Table 3), which is similar to that found in other tropical and temperate continental shelf sediments^5^,^15^.

The total microbial C mineralization obtained from fluxes can be explained fully by DENIT and FeR in September (Table 3). However, a deficit of 9 and 15 mmol m^−2^ d^−1^ in February at St 5 and St 6 after correcting for DENIT and FeR (Table 3) indicates seasonal differences in organic matter reactivity and availability between February and September. Experimental evidence suggests that labile organic matter is mineralized with similar rates regardless of redox conditions, whereas refractory organic matter degrades faster under oxic than anoxic conditions^29^. Lower availability of labile organic matter (e.g. chlorophyll-a) and more refractory and O_2_-sensitive organic matter could therefore explain the low jar-based C mineralization in February^3^,^27^. The unexplained deficit in microbial C mineralization may be also related to higher contribution of aerobic respiration (39–60%) in February (Table 3) as indicated by higher O_2_ levels in the water and extended thickness of the oxidized zone from 0.1 mm (September) to 2–3 mm (February). This is probably coupled to the much higher fauna abundance that drives faster downward transport of O_2_ through bioirrigation and thus faster degradation and depletion of labile organic matter.

The large difference in redox profiles and electron acceptor partitioning between September and February suggests that the area is subjected to strong seasonal variation in sediment biogeochemistry. This change may be associated with the upwelling and cold-front events that shift water column conditions and phytoplankton composition dramatically and in turn alters the organic matter reactivity as well as the structure of macrobenthic communities^14^,^30^. The low O_2_ in the water column and sediment during the sampling in September can be explained by the windy conditions causing wash out of benthic fauna due to particle resuspension and high content of labile organic matter associated to regenerated primary production^31^. The generally calm conditions during late summer in February allow the re-establishment of O_2_ to higher levels and increased density of benthic fauna. However, the cascading effects of upwelling and cold-fronts on microbial metabolic pathways are probably strongly controlled by the duration and exact time of the year that these events occur. Upwelling periods can happen from November to March, when high nitrate levels in the water stimulate diatom production^8^,^14^. Windy cold-fronts dominate the rest of the year, causing sediment resuspension and nutrient release to the water, usually leading to blooms of phytoflagellates^8^,^14^. Therefore, large inter-annual variations can be observed^13^ and care should be taken when comparing the results of this study with other shallow marine areas.

In summary, this study shows that FeR is the major pathway of anaerobic C mineralization in Ubatuba Bay sediments, SE Brazil. A similar dominant role of FeR for anaerobic C mineralization has never been documented before for coastal Brazilian sediments. Contrasting redox conditions between winter and summer reflect differences in the partitioning of aerobic and anaerobic processes as a consequence of changing environmental conditions (i.e. upwelling, cold fronts), which in turn influences organic matter reactivity, macrobenthic communities and bioturbation. Bioturbation of small benthic fauna is essential for oxidizing the sediment in summer, while large-sized species stimulates the reoxidation of reduced compounds, e.g. Fe(II), throughout the year.

## Methods

### Study site and sampling

The study was performed in September 2012 and February 2014, corresponding to winter and summer, respectively, in Ubatuba Bay. Ubatuba is located on the coast of Sao Paulo State, Southeastern Brazil, on the transition between tropical and subtropical climate zones. A mountain range “Serra do Mar” with altitudes up to 1000 m reaches almost all Ubatuba shores, forming small enclosed bays and influencing the geological composition of coastal sediments, which consists mainly of granite and migmatite minerals^20^,^21^.

The area is considered meso-oligotrophic with phytoplankton biomass ranging from 1 to 3 mg m^−3^ 30. The Ubatuba coast is in spring-summer affected by upwelling of cold and nutrient rich South Atlantic Coastal Water (SACW), which may lead to diatom blooms. In rest of the year, the warmer Coastal Water (CW) dominates with frequent cold front events (ca. 4 per month) and S-SE waves that lead to sediment resuspension, nutrient release and stimulated phytoflagellate blooms^8^,^32^. Benthic macrofauna typically consists of high numbers of small-sized species varying from 0.1 to 3.0 mg AFDW ind^−1^ 14,24. Larger-sized macrofaunal species are less frequent, and include, the bivalve *Tellina* sp., burrow dwelling Enteropneusta sp. (Hemichordata), the sipunculid *Thysanocardia catarinae*, and the ophiuroids *Amphiodia atra* and *Hemipholis elongata*^14^,^22^.

Intact large sediment cores (10 cm i.d.) were collected with a multicorer from the research vessel “Veliger II”, Sao Paulo University. Sampling was conducted at three stations St 5, St 6, and St 7, positioned along an increasing depth gradient from 5 to 12 m in Ubatuba Bay^14^. The bottom water temperature was 22–23° C in September 2012 and 22–26° C in February 2014, with the highest temperatures recorded at the shallow St 5. A total of 3 × 3 cores were collected at each station for: (1) flux incubations, sediment characteristics, bioirrigation, and porewater analysis; (2) macrofauna analysis; and (3) anoxic sediment (jar) incubations. The three replicate cores were subsampled with smaller cores having 8 cm i.d. to fit the experimental set-up for flux incubations. The three cores for macrofaunal analysis were sieved on board through a 500 μm mesh and retained material was preserved in 70% ethanol. The last three cores for anoxic sediment incubations were sliced in the laboratory for “jar” preparations.

### Benthic metabolism

Triplicate cores (8 cm i.d.) from St 5, St 6 and St 7 were transferred to a 90 L tank containing seawater with salinity of 35 and maintained in the laboratory in darkness at 22° C. Water stirring was assured by a central motor that rotated magnetic bars placed in the headspace of individual sediment cores. Three sequential flux measurements were done 1, 2–3 and 6 days after core sampling by incubating the sealed cores for 4–8 h with constant stirring. Water samples for O_2_, TCO_2_ (=H_2_CO_3_ + HCO_3_^−^ + CO_3_^2−^), NH_4_^+^, NO_x_^–^ (=NO_3_^−^ + NO_2_^−^), and PO_4_^3−^ were collected at the start and end of flux incubations. O_2_ samples were immediately fixed with Winkler reagents. TCO_2_ samples were stored at 4° C in gas-tight vials and a sample for nutrients was stored at −20° C. O_2_ concentrations were determined by Winkler titrations^33^ and TCO_2_ was determined by flow injection analysis^34^. Nutrients were analyzed on a Lachat Quickchem 8500 auto-analyzer. Fluxes were calculated from the concentration change of O_2_, TCO_2_ and nutrients in the headspace of individual cores.

### Redox profiles

Two vertical redox profiles were measured on each of two random cores from each station with a redox needle electrode (RD-N, Unisense A/S) coupled to a calomel reference electrode and an Impo Electronic type 1510 pH/mV-meter. Measurements were undertaken stepwise at high resolution (0.5–1 mm) until ca. 1 cm depth and thereafter in 2–5 mm steps, until ca. 3 cm sediment depth. The signal was allowed to stabilize for 3 min before moving the electrode to the next sediment depth. The measured values were corrected for the use of calomel electrode according to: Eh = E_measured_ + 244 mV.

### Porewater analysis

After flux incubations, the cores were sliced into the intervals 0–1, 1–2, 2–4, 4–6, 6–8, 8–10, 12–14 and 16–18 cm. Sediment slices were homogenized and subsamples were transferred to 50 mL centrifuge tubes and centrifuged at 500 × *g* for 15 min. Supernatant porewater was GF/C filtered and subsampled for different chemical analysis. One 500 μL subsample was fixed with saturated HgCl_2_ (volume ratio of 9:1) and stored at 4° C for TCO_2_ analysis as described above. Another subsample was preserved with 0.5 M HCl (volume ratio of 4:1) for dissolved iron (Fe^2+^) and analyzed by the ferrozine color reaction method^35^. A pooled subsample for SO_4_^2−^, Br^−^ and nutrient analysis was stored frozen at −20° C. SO_4_^2−^ and Br^−^ were analyzed by ion chromatography (Dionex ICS-2000) and normalized to chloride concentrations. Nutrients were analyzed as mentioned above.

### Solid-phase sediment analysis

Subsamples of wet sediment were collected for water content, density and porosity. Water content was determined as weight loss of sediment after drying (24 h at 105° C). Wet density was determined as weight of a known volume of sediment using 5 mL cut-off syringes. Sediment grain size was analyzed following the Wentworth scales of sieving and size classification, and TOC and TN were measured after removal of carbonates by acidification on a Leco CNS 2000 Elemental Analyzer^14^.

Chlorophyll-a and phaeopigments were measured on the 0–1, 1–2 and 2–3 cm sediment intervals after extracting ca. 500 mg wet sediment subsamples in 5 mL of 90% ethanol in darkness at 4° C for ca. 24 h. The test tubes were centrifuged at 500 × *g* for 15 min and the absorbance of supernatants was measured at 665 and 750 nm before and after acidification (10% HCl)^36^. Other wet sediment subsamples (ca. 300 mg) were transferred immediately after core slicing to centrifuge tubes containing 5 mL of 0.5 M HCl for solid-phase iron analysis^37^. The centrifuge tubes were shaken for 30 min and subsequently centrifuged at 500 × *g* for 15 min. Reactive Fe(II) was analyzed in the extract by the ferrozine method as described above. Total reactive solid phase iron (TRFe) was measured in the extract by the ferrozine method after reduction with hydroxylamine. Reactive Fe(III) was calculated from the difference between TRFe and reactive Fe(II).

### Bioirrigation

One day before core slicing the seawater in the tank was enriched with NaBr to 7–13 mM. The following day porewater samples were obtained during core slicing as described above and analyzed for Br^−^. Total community bioirrigation (L m^−2^ d^−1^) was calculated as the difference between measured porewater inventories of Br^−^ and the estimated molecular Br^−^ diffusion into the sediment based on incubation time, Br^−^ concentration in overlying water and sediment porosity. Profiles of molecular Br^−^ diffusion were estimated by one-dimensional diagenetic modeling using diffusion coefficients corrected for porosity^38^.

### Macrofauna analysis

After sorting, all macrofauna was identified and classified into functional bioturbation groups: epifaunal, surface and gallery diffusors, and upward and downward conveyors^2^. Wet weight of higher taxonomic groups of macrofauna (i.e. polychaetes, bivalves, gastropods, ophiuroids etc.) was converted to AFDW^39^,^40^. Macrofauna O_2_ consumption q (μL O_2_ h^−1^) was calculated for average individual ash free dry weight biomass (W) according to equation for poikilotherms^41^: log q = 1.29 + 0.74 log W at 20° C, and adjusted to the incubation temperature using Q_10_ = 2.

### Anoxic sediment incubations

Three large cores (10 cm i.d.) from each station were sliced into depth intervals 0–2, 4–6, 8–10 and 16–18 cm. Sediment from each interval was pooled and homogenized, and transferred to eight 50 mL centrifuge tubes (jars) leaving no headspace^27^. Jars were sealed gas tight and buried in anoxic sediment to avoid oxygen intrusion and incubated in darkness at 22° C for 10–22 days. Two jars from each interval were sacrificed every 2–4 days by centrifugation at 500 × *g* for 15 min. The supernatant porewater was GF/C filtered and separated into subsamples for TCO_2_, SO_4_^2−^, dissolved iron (Fe^2+^), and nutrients as described above. In February 2014, iron reduction was estimated based on solid-phase 0.5 M HCl Fe extraction. The jars used for this approach were opened before centrifugation and ca. 300 mg wet sediment were collected for extraction of solid-phase Fe as described above. NH_4_^+^ production rates were corrected for adsorption. Linear NH_4_^+^ adsorption coefficients were determined on wet sediment subsamples after incubation with different NH_4_^+^ concentrations and analysis with and without addition of 2 M KCl^42^.

### Statistical analysis

Differences among the factors stations (St 5, St 6 and St 7), time (September and February) and interaction time x station were tested by two-way ANOVA for flux rates, pigments content, macrofaunal density, and bioirrigation rates. Porewater and microbial rate profiles were tested using depth integrated values (20 cm depth). The normality of data was checked by Shapiro-Wilk test. Pairwise *post hoc* comparisons among stations and between sampling dates were done by applying Tukey tests. All statistical analyses were performed with a significance level α=0.05 using the software SigmaPlot 11.2.

## Additional Information

**How to cite this article**: Quintana, C. O. *et al.* Carbon mineralization pathways and bioturbation in coastal Brazilian sediments. *Sci. Rep.* **5**, 16122; doi: 10.1038/srep16122 (2015).

## Supplementary Material

Supplementary Information

## Figures and Tables

**Table 1:** Exchange fluxes of TCO_2_, O_2_, NH_4_^+^, and NO_X_^−^ across the sediment-water interface in September 2012 and February 2014 at St 5, St 6 and St 7.

	St 5	St 6	St 7
TCO_2_ (mmol m^−2^ d^−1^)			
September 2012	23.1 ± 2.1	17.2 ± 2.0	18.8 ± 3.2
February 2014	31.3 ± 3.4	35.0 ± 3.4	33.9 ± 3.5
O_2_ (mmol m^−2^ d^−1^)			
September 2012	−9.2 ± 0.6 (2.7)	−12.1 ± 0.7 (1.5)	−11.5 ± 1.6 (1.8)
February 2014	−32.7 ± 1.8 (1.0)	−31.7 ± 1.5 (1.1)	−35.4 ± 3.1 (1.0)
NH_4_^+^ (mmol m^−2^ d^−1^)			
September 2012	−0.1 ± 0.1	−0.5 ± 0.2	−1.2 ± 0.7
February 2014	1.1 ± 0.3	1.2 ± 0.6	1.5 ± 0.7
NO_X_^−^ (mmol m^−2^ d^−1^)			
September 2012	−0.6 ± 0.2	−0.4 ± 0.1	−0.4 ± 0.2
February 2014	−0.1 ± 0.05	0.2 ± 0.1	−0.1 ± 0.04

Values represent mean ± standard error (n = 9). Values in parenthesis represent RQ (=CO_2_ efflux/O_2_ consumption).

**Table 2:** Abundance of macrofauna, total community O_2_ respiration and bioirrigation rates in September 2012 and February 2014 at St 5, St 6 and St 7.

	St 5	St 6	St 7
Macrofauna abundance (ind m^−2^)			
September 2012	92 ± 92	138 ± 80	184 ± 46
February 2014	1537 ± 267	1777 ± 267	1297 ± 381
Macrofauna biomass (mg ind m^−2^)			
September 2012	144 ± 144	402 ± 232	32 ± 8
February 2014	264 ± 46	245 ± 37	3029 ± 890
Community O_2_ consumption (mmol m^−2^ d^−1^)			
September 2012	3.1	7.5	1.2
February 2014	8.2	9.6	10.5
Community bioirrigation (L m^−2^ d^−1^)			
September 2012	4.4 ± 0.1	9.5 ± 7.0*	4.5 ± 1.0
February 2014	12.0 ± 4.3*	13.6 ± 0.4	7.6 ± 0.0*
*Bioirrigation excluding large fauna (L m^−2^ d^−1^)			
September 2012	—	2.9 ± 0.8	—
February 2014	7.8 ± 1.7	—	5.5 ± 0.0

*Bioirrigation rates excluding large fauna. Values represent mean ± standard error (n = 3).

**Table 3:** Partitioning of C mineralization processes in September 2012 and February 2014 at St 5, St 6 and St 7.

Pathways of C mineralization	St 5	St 6	St 7
Total (fauna + microbial) (mmol C m^−2^ d^−1^)			
September 2012	23 ± 2	17 ± 2	19 ± 3
February 2014	31 ± 3	35 ± 3	34 ± 3
Microbial (−fauna metab.) (mmol C m^−2^ d^−1^)			
September 2012	20 ± 2	11 ± 2	18 ± 3
February 2014	23 ± 3	25 ± 3	23 ± 3
Anaerobic C mineralization (mmol C m^−2^ d^−1^)			
September 2012	25 ± 4	12 ± 3	22 ± 3
February 2014	11 ± 2	8 ± 2	23 ± 4
Fe reduction (%)			
September 2012*	(81)	(76)	(73)
February 2014	(48)	(32)	(61)
Denitrification (%)			
September 2012	(19)	(24)	(27)
February 2014	(13)	(9)	(5)
Other anaerobic process (%)			
September 2012	—	—	—
February 2014	—	—	(34)
Excess O_2_ respiration (%)			
September 2012	—	—	—
February 2014	(39)	(60)	—

*Based on estimated Fe reduction rates: see text for explanation. Values represent mean ± standard error (n = 3). Values in parenthesis represent relative contribution to total microbial C mineralization (i.e. TCO_2_ effluxes minus fauna metabolism).
