# Supplementary material for: Carbon mineralization pathways and bioturbation in coastal Brazilian sediments
Source: Sci Rep. 2015 Nov 3;5:16122. doi: 10.1038/srep16122 (PMC4630785; doi:10.1038/srep16122)
Supplement: Supplementary Information [file srep16122-s1.pdf]

Supplementary information

Carbon mineralization pathways and bioturbation in coastal Brazilian sediments

Cintia O. Quintana[1,2*], Maurício Shimabukuro[1], Camila O. Pereira[1], Betina G. R. Alves[1], Paula C. Moraes[1], Thomas Valdemarsen[2], Erik Kristensen[2], Paulo Y. G. Sumida[1]

1. Instituto Oceanográfico da Universidade de São Paulo, Praça do Oceanográfico, 191, Cidade Universitária, 05508-120, São Paulo, Brazil

2. Department of Biology, University of Southern Denmark, Campusvej 55, Odense M, 5230, Denmark

* Corresponding author: cintiaorgano@gmail.com

Table S1: Summary of statistical analysis from variables with significant differences. Letters “a”, “b” and “c” represent significant difference within the factors “time” and “station” observed in Tukey post hoc tests.

Two-way ANOVA	F	p	Tukey test				
**Bottom water O_2**			September	February	St 5	St 6	St 7
Time	$F_{1,16} = 393.77$	<0.001	a	b			
Station	$F_{1,16} = 0.70$	0.515					
Time X Station	$F_{1,16} = 0.90$	0.431					
**Sediment Chlorophyll-a**							
Time	$F_{1,16} = 19.36$	0.001	a	b	a	b	bc
Station	$F_{1,16} = 13.11$	0.001					
Time X Station	$F_{1,16} = 6.69$	0.013					
**Sediment Phaeopigments**							
Time	$F_{1,16} = 21.33$	<0.001	a	b			
Station	$F_{1,16} = 1.06$	0.380					
Time X Station	$F_{1,16} = 2.41$	0.136					
**TCO_2 efflux**							
Time	$F_{1,56} = 31.61$	<0.001	a	b			
Station	$F_{1,56} = 0.53$	0.591					
Time X Station	$F_{1,56} = 1.98$	0.149					
**O_2 flux**							
Time	$F_{1,56} = 319.05$	<0.001	a	b			
Station	$F_{1,56} = 1.17$	0.316					
Time X Station	$F_{1,56} = 1.34$	0.270					
**NO_x flux**							
Time	$F_{1,36} = 118.17$	<0.001	a	b	a	b	a
Station	$F_{1,36} = 7.66$	0.002					
Time X Station	$F_{1,36} = 1.36$	0.272					

Continuation of Table S1

Two-way ANOVA	F	p	Tukey test				
			September	February	St 5	St 6	St 7
**Macrofaunal abundance**							
Time	$F_{1,17} = 48.55$	<0.001	a	b			
Station	$F_{1,17} = 0.60$	0.563					
Time X Station	$F_{1,17} = 1.05$	0.379					
**Macrofaunal biomass**							
Time	$F_{1,17} = 6.28$	0.028	a	b			
Station	$F_{1,17} = 0.85$	0.453					
Time X Station	$F_{1,17} = 3.47$	0.065					
**Macrofaunal bioirrigation**							
Time	$F_{1,15} = 13.57$	0.004	a	b			
Station	$F_{1,15} = 0.63$	0.555					
Time X Station	$F_{1,15} = 1.38$	0.296					
**Surface diffusors**							
Time	$F_{1,17} = 11.84$	0.005	a	b			
Station	$F_{1,17} = 0.75$	0.493					
Time X Station	$F_{1,17} = 1.05$	0.381					
**Gallery diffusors**							
Time	$F_{1,17} = 17.80$	0.001	a	b			
Station	$F_{1,17} = 0.13$	0.88					
Time X Station	$F_{1,17} = 0.13$	0.88					
**Epifaunal diffusors**							
Time	$F_{1,17} = 17.80$	0.001	a	b			
Station	$F_{1,17} = 0.13$	0.88					
Time X Station	$F_{1,17} = 0.13$	0.88					

Table S2: Stoichiometry of carbon and nitrogen in porewater and jars and N mineralization processes at St 5, St 6 and St 7 during September 2012 and February 2014.

	St 5	St 6	St 7
**C:N in porewater**			
September 2012	7.2	7.8	5.5
February 2014	6.0	7.3	8.8
**C:N in jars***			
September 2012	8.2	8.5	7.7
February 2014	6.4	7.7	9.4
**Total N mineralization (mmol m^{-2} d^{-1})**			
September 2012	2.4	1.3	2.3
February 2014	3.6	3.2	2.4
**NH_4^+ production jars * (mmol m^{-2} d^{-1})**			
September 2012	3.0	1.4	2.8
February 2014	1.8	1.1	2.4
**Nitrification (mmol m^{-2} d^{-1})**			
September 2012	2.5	1.8	3.5
February 2014	2.5	2.0	0.9
**Denitrification (mmol m^{-2} d^{-1})**			
September 2012	3.1	2.2	3.9
February 2014	2.6	1.8	1.0

* Corrected for NH_4^+ adsorption. C:N in porewater was determined by the slope of linear regression between TCO_2 and NH_4^+ in the upper 10 cm of the sediment. Total N mineralization was determined by dividing the TCO_2 effluxes with the C:N in jars. Nitrification was estimated by subtracting NH_4^+ fluxes from the total N mineralization. Denitrification was estimated by nitrification minus NO_x^- fluxes.
